# Surgery for scar revision and reduction: from primary closure to flap surgery

**DOI:** 10.1186/s41038-019-0144-5

**Published:** 2019-03-01

**Authors:** Rei Ogawa

**Affiliations:** Department of Plastic, Reconstructive and Aesthetic Surgery, Nippon Medical School, 1-1-5 Sendagi Bunkyo-ku, Tokyo, 113-8603 Japan

**Keywords:** Scar, Keloid, Hypertrophic scar, Scar contracture, Surgery, Z-plasty, W-plasty, Local flap, Expander, Perforator flap

## Abstract

Scars are the final result of the four processes that constitute cutaneous wound healing, namely, coagulation, inflammation, proliferation, and remodeling. Permanent scars are produced if the wounds reach the reticular dermis. The nature of these scars depends on the four wound healing processes. If the remodeling process is excessive, collagen degradation exceeds collagen synthesis and atrophic scars are produced. If the inflammation phase is prolonged and/or more potent for some reason, inflammatory/pathological scars such as keloids or hypertrophic scars can arise. If these pathological scars are located on joints or mobile regions, scar contractures can develop. When used with the appropriate timing and when selected on the basis of individual factors, surgical techniques can improve mature scars. This review paper focuses on the surgical techniques that are used to improve mature scars, burn scars, and scar contractures. Those methods include z-plasties, w-plasties, split-thickness skin grafting, full-thickness skin grafting, local flaps (including the square flap method and the propeller flap), and expanded flaps, distant flaps, regional flaps, and free flaps.

## Background

Cutaneous wound healing involves four overlapping sequential stages, namely, coagulation, inflammation, proliferation, and remodeling. Scars are the endpoint of these processes. Whether significant scarring occurs seems to depend on the depth of the cutaneous wounds: when Dunkin et al.[1] subjected normal human skin to elective linear incisions of variable depth, they found that permanent scarring did not occur when the skin injury was less than 0.57 mm deep (i.e., about a third of the thickness of the dermis). However, deeper dermal injuries did result in permanent scars. Thus, permanent scars are only produced if wounds reach the reticular dermis.

Deep cutaneous wounding disrupts the normal structure of the reticular dermis [2]. During wound healing, this tissue is replaced by collagen, which is produced by fibroblasts in the proliferation phase: this is known as granulation tissue. Thereafter, during the remodeling phase, collagen synthesis becomes matched by collagen degradation, the granulation tissue is reorganized, and stronger collagen is laid down, thereby generating the mature scar. The nature of this scar is shaped by the four wound-healing processes. For example, if the remodeling phase is excessive, collagen degradation exceeds collagen synthesis and atrophic scars are produced. Moreover, if the preceding inflammation phase is prolonged and/or more potent for some reason, inflammatory/pathological scars such as keloids or hypertrophic scars form. Notably, if these pathological scars are located on joints or mobile regions, they can develop into scar contractures.

This review paper focuses on the surgical techniques that are used to improve mature scars, burn scars [3], and scar contractures. It should be noted here that while the methods discussed in this paper can be used to treat pathological scars such as keloids [4], these methods must be accompanied by postoperative adjuvant therapies; otherwise, there is a large risk of scar recurrence.

## Review

### Z-plasty or w-plasty incisions and primary suturing

**Fig. 1:**
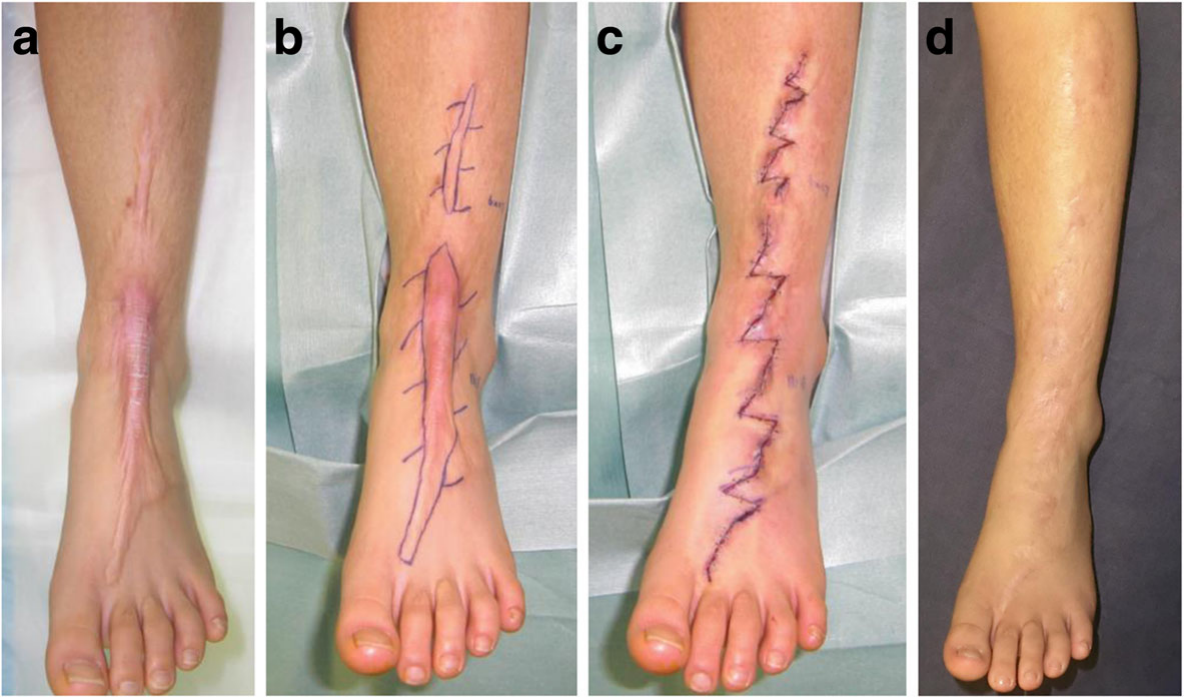
Reconstruction of scar contractures by using multiple z-plasties. **a** Preoperative view. **b** Design of the z-plasties. **c** Immediate postoperative view. **d** Eighteen months after surgery. A major benefit of z-plasties is that segmented scars mature faster than long linear scars

Zig-zag incision and suture strategies, including z-plasty and w-plasty, are good for releasing linear scar contractures and tension [5, 6]. A major benefit of z-plasties is that segmented scars mature faster than long linear scars (Fig. 1). This is because vertical scars that run along the long axis of the body part are placed under greater skin tension than horizontal scars that lie perpendicular to the long axis. This tension prolongs the inflammatory stage of wound healing, which in turn causes scar hypertrophy. Interspersing vertical scars with horizontal scars disrupts the tension on the vertical scars, thereby ensuring the rapid switch from the inflammatory phase of wound healing to the proliferative and remodeling phases. This also explains why zig-zag incision and suturing significantly reduces the risk of pathological scar development when a scar is located on a joint. Ideally, the triangular flaps of the z-plasty should not include scars. This is because while healthy skin extends readily after surgery, thereby effectively releasing tension, scarred skin is much less extensible. Moreover, inclusion of scarred tissue in the flaps increases the risk that the edges of the flaps become necrotic.

**Fig. 2:**
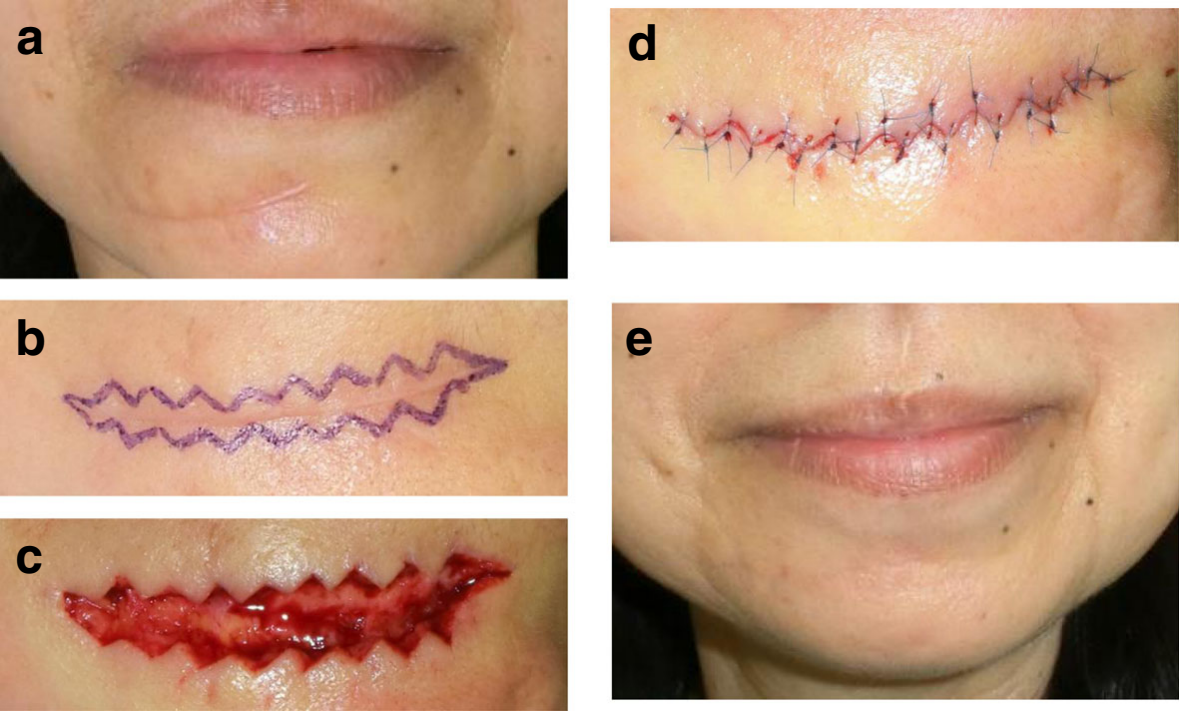
Resection and w-plasty of a scar between the lower lip and the jaw. **a** Preoperative view. **b** Design of the w-plasty. **c** Intraoperative view. **d** Immediate postoperative view. **e** Twelve months after the operation. The indication for w-plasty is a scar on the flat surfaces of the face such as the cheek and the area between the lower lip and the jaw

The main advantage of the w-plasty is the broken line effect, namely, the fact that zig-zag scars are less visible because they reflect light more poorly than linear scars. Consequently, the indication for w-plasties is a scar on flat surfaces of the face such as the cheek or the area between the lower lip and the jaw (Fig. 2). However, z-plasties are more suitable for scars on the forehead and nasolabial area because it is easy to design a z-plasty so that the incision line matches the skin creases or folds in these areas. W-plasties are not suitable for scars on major joints such as the axilla and elbow.

### Skin graft

**Fig. 3:**
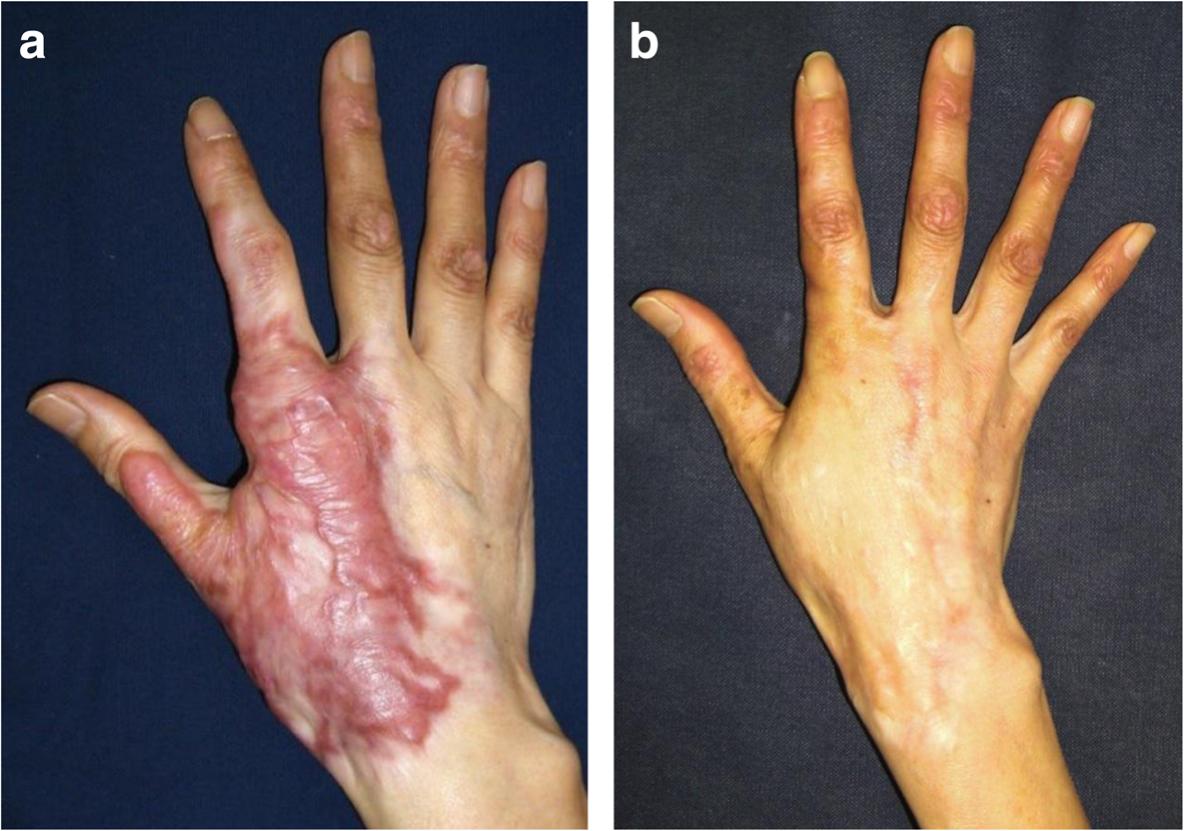
Full-thickness skin graft for hypertrophic scars on the hand. **a** Preoperative view. **b** Eighteen months after the operation. Full-thickness skin grafts should be the first choice for secondary scar reconstruction. (The figure is reproduced with permission from the article [7] (Copyright 2010 by Wolters Kluwer Health))

Skin grafts are useful for replacing particularly large scars. Powered dermatomes allow the surgeon to rapidly harvest large areas of the skin that can then be used to cover large recipient sites, including primary wounds and the wounds left after burn scars are removed for reconstruction. However, these split-thickness skin grafts (STSG) tend to develop severe secondary contractures and should therefore be followed by secondary scar reconstruction with full-thickness skin grafts (FTSG), which are much less prone to such contractures (Fig. 3) [7]. However, FTSGs survive less well than STSGs because of the increased diffusion distance and the longer time that these grafts need before they achieve complete revascularization.

Skin grafts can be fixed with negative pressure wound therapy devices as well as with the traditional gauze tie-over method [8]. We have also used external wire frame fixation for skin grafts, particularly for those on the eyelid, the perioral area, and the digital joints [9].

**Fig. 4:**
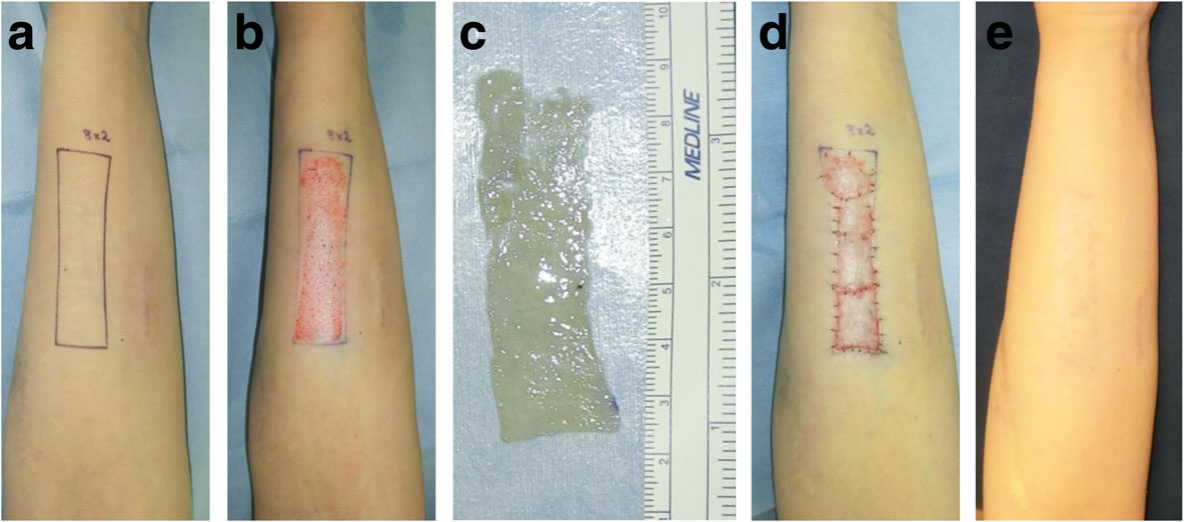
Thin split-thickness skin graft for self-harm scars on the forearm. **a** The scar area. **b** After the thin skin graft was harvested. **c** The harvested thin skin. **d** Immediate postoperative view. **e** Twelve months after the operation. A thin split-skin graft (8/1000 in.) was taken from the affected area, after which the wide dermal scars were excised and the graft was closed by placing the graft back onto the harvest site

In recent years, we presented a new surgical approach for the revision of deliberate self-harm scars [10]. This approach is called the isotopic skin graft technique and it involves harvesting a thin STSG (6–8/1000 in.) from the affected area, excising the wide dermal scar, and then placing the graft back onto the harvest site. The graft is so thin that it does not include the reticular dermis. This ensures that hypertrophic scars will not develop (Fig. 4).

### Local flaps

**Fig. 5:**
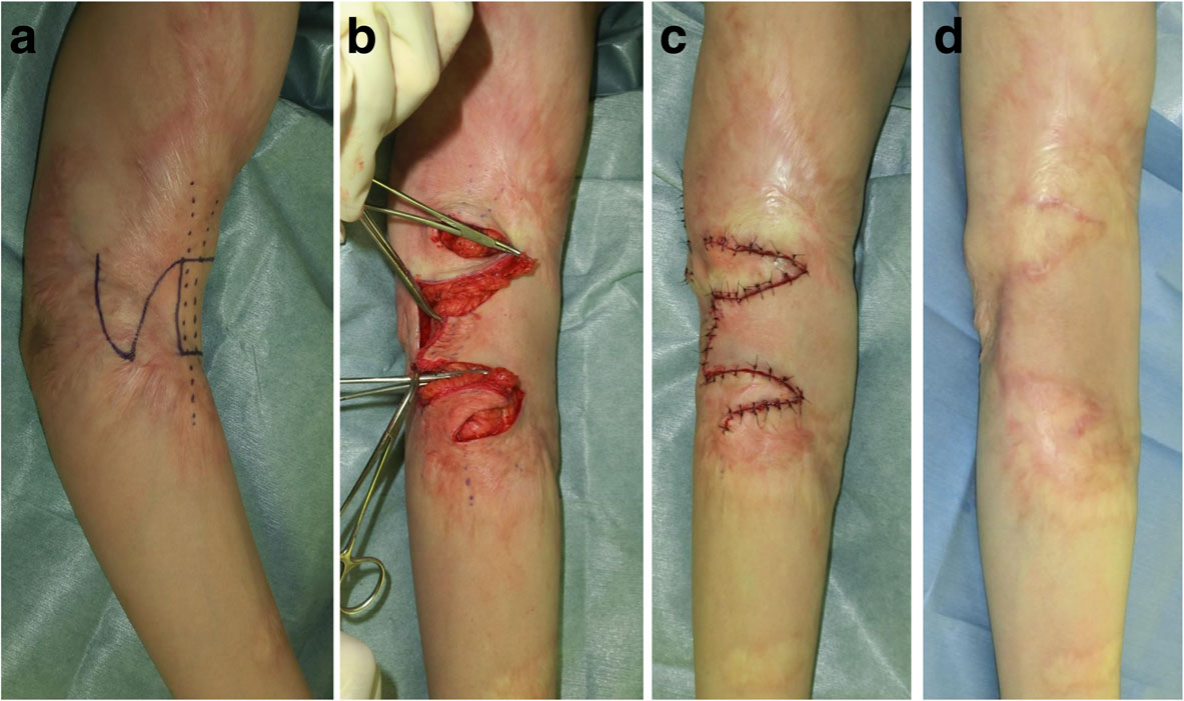
The square flap method for elbow joint contracture. **a** Design of the square flap method. **b** Intraoperative view. **c** Immediate postoperative view. **d** Eighteen months after the operation. The square flap method involves one square flap and two triangular flaps. The extensibility of these flaps is much higher than that of the triangular z-plasty flaps

Various local flaps are useful for releasing scar contractures. Moreover, because local flaps expand naturally after surgery, they are not prone to postsurgical contractures. By contrast, skin grafts do not expand, which means that skin grafting tends to generate secondary contractures that result in circular pathological scars around the grafted skin. Local flaps can be traditionally classified as advancement flaps, rotation flaps, and transposition flaps. These flaps should preferably have skin pedicles because although it is technically easier to transfer island flaps to the recipient site than skin-pedicled flaps, they release contractures less effectively. The postoperative extensibility of the flap should be considered when determining which flap design is optimal for the individual patient. With regard to the skin-pedicled flaps, the square flap [11] method is particularly useful for reconstructing major joint scar contractures because these flaps can theoretically extend by threefold (Fig. 5).

**Fig. 6:**
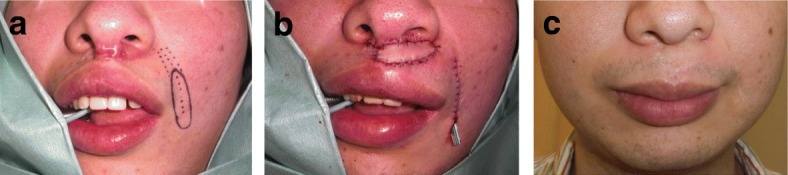
The propeller flap method for upper lip scar contracture. **a** Design of the propeller flap approach. **b** Immediate postoperative view. **c** Thirty-six months after the operation. In this case, a nasolabial flap was harvested to serve as the tunneled propeller flap

When burn injuries are extensive, it can be difficult to design traditional flaps. In this case, propeller flaps can be an excellent alternative. The original propeller flap [12] uses intact skin in the fossa of the elbow or axilla that is elevated on a central subcutaneous pedicle. This flap can be applied to burn contractures on the upper lip (Fig. 6) and other sites as long as it can be harvested as a perforator-pedicled propeller flap [13].

### Expanded flaps

**Fig. 7:**
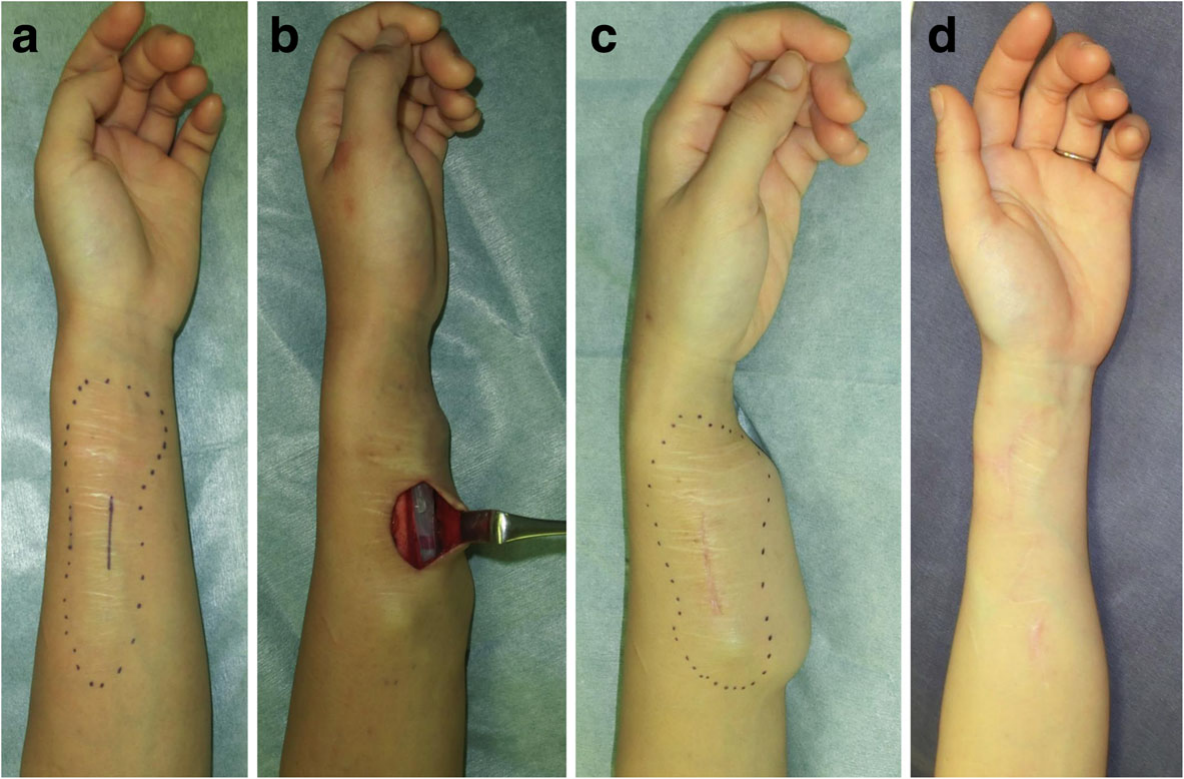
The expander flap method for forearm scars. **a** Design of the first operation, where an expander was implanted. **b** View during the first operation. **c** View immediately before the second operation. **d** Eighteen months after the second operation. Healthy skin was expanded for 3 months after the first operation. In the second operation, the entire scar area was excised and closed primarily with z-plasties.

Expanded healthy skin near the recipient site is the ideal material for scar reconstruction because it matches the recipient site in terms of both color and texture (Fig. 7). For example, if the scars are on the anterior neck, expanders can be implanted into the anterior chest wall. Moreover, if the scars are on the forearm, several small expanders can be implanted simultaneously: where they are located and how many are used depends on the size and shape of the scars and the remaining healthy skin. One disadvantage of expanded flap surgery is that two operations are needed. Moreover, the patient must return repeatedly to the hospital for the saline injections into the expander. However, the latter disadvantage has been largely solved by the recent development of new expanders such as the AirXpanders [14], where the device expands by remote-controlled release of compressed CO_2_.

### Distant flaps

**Fig. 8:**
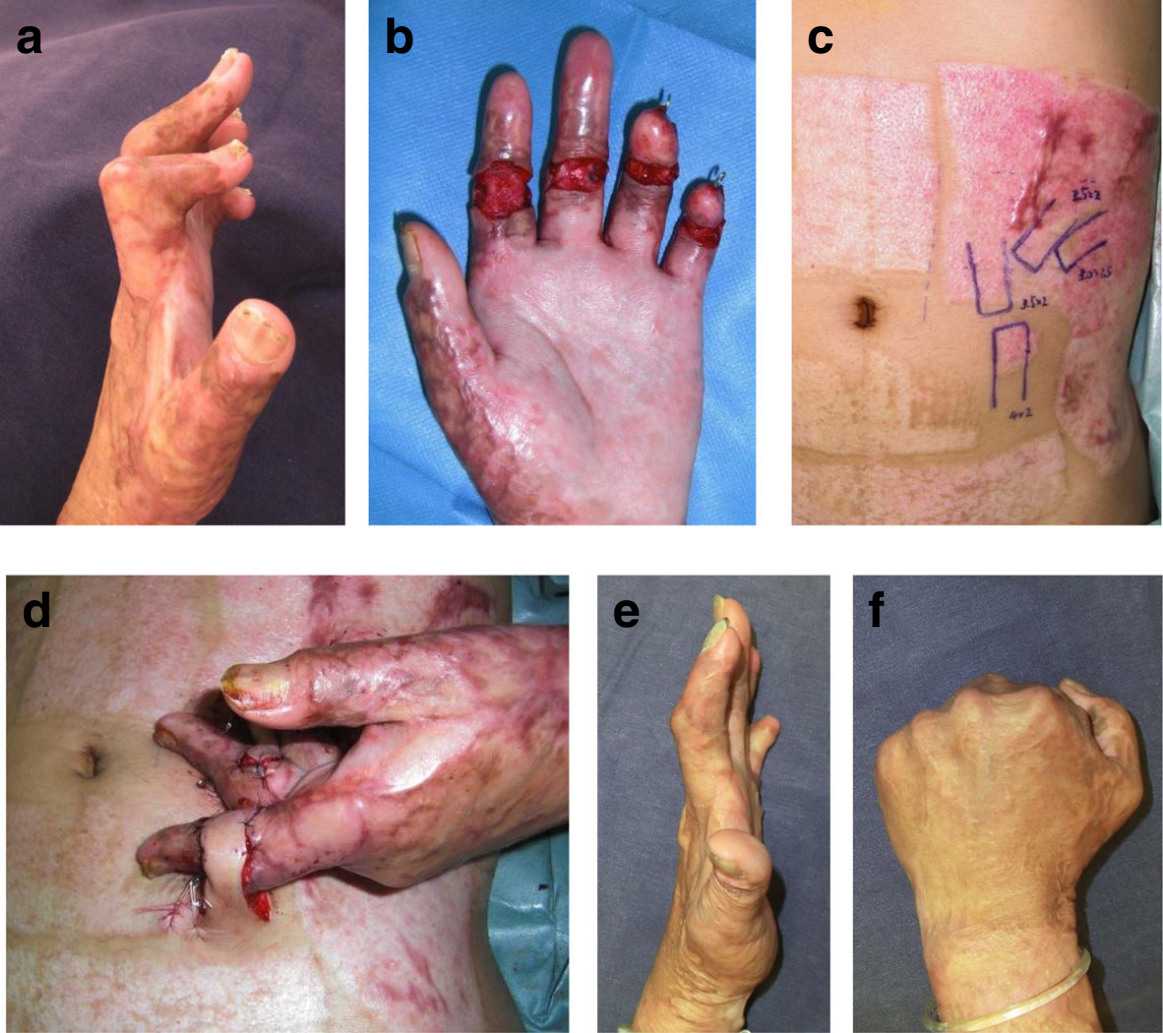
Reconstruction of multiple finger joint contractures with distant flaps. **a** Preoperative view. **b** View after releasing the contractures. **c** Design of the abdominal distant flaps. **d** Immediately after the operation. **e**, **f** Thirty-six months after the operation. Multiple finger joint contractures were reconstructed by using abdominal distant flaps. The flaps were transplanted and cut 3 weeks after the operation in this case

In recent years, the indications for distant flaps have dropped because of the development of free tissue transfer under a microscope. However, we believe that distant flaps are still useful. We recently reconstructed multiple finger joint contractures by using abdominal distant flaps (Fig. 8). The flaps were transplanted and then cut 2–3 weeks after the operation. One disadvantage of this method is that the patient may feel some discomfort during this interim period. However, the advantage of the method is that it is less invasive than other methods because it allows preservation of the recipient blood vessels.

### Regional flaps

Regional flaps should be considered for the reconstruction of extensively burned contractures on movable areas such as the anterior neck, axilla, and joints to prevent re-contracture by grafted skin.

For the anterior neck, the pectoralis major muscle flap [15], the latissimus dorsi flap [16], and the trapezius muscle flap [17] can be used as pedicled regional flaps. Many perforator flaps are available, including the supraclavicular flap [18], the internal mammary artery perforator (IMAP) flap [19], and the superficial cervical artery perforator (SCAP) flap [20].

For the axilla [21], traditional regional flaps that can be used include the scapular flap, the para-scapular flap, the circumflex scapular artery propeller flap, the latissimus dorsi muscle flap [16], and the thoracodorsal artery perforator (TAP) flap [22].

**Fig. 9:**
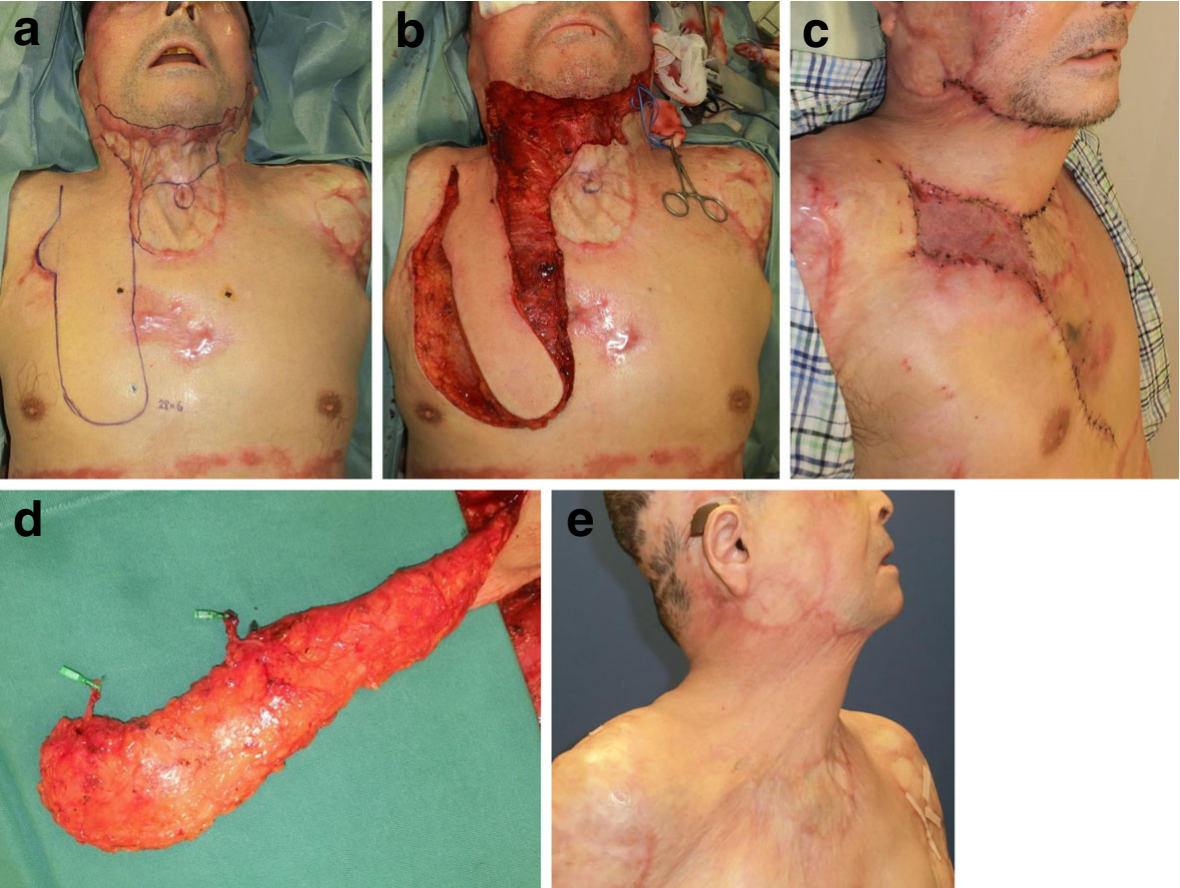
Internal mammary artery supercharged-transposition flap for anterior neck scar contractures. **a** Design of the flap. **b** Intraoperative view. **c** Perforators were attached to the flap. **d** View 1 week after surgery. **e** Eighteen months after the operation. A transposition flap bearing the internal mammary artery perforator was harvested from the anterior chest wall to repair the neck wounds that were left after removing the scar. At the same time, the tracheal fistula was covered by the flap. The esthetic and functional results were acceptable. (The figure is reproduced with permission from the article [19] published by Wolters Kluwer Health (Copyright 2018 by Rei Ogawa et al.)) In case of PRS-GO article, copyright is kept by authors

The angiosome [23] and perforasome [24] concepts mean that a large number of flaps are available. Which of these flaps are most suitable for individual patients depends on the size of the perforator as well as the geometry and size of the flap that is to be transferred. If a large flap is needed for reconstruction, supercharged vessels can be attached to the distal area of the regional flap and then anastomosed with recipient vessels. Moreover, we have used perforator-supercharged “super-thin flaps” for anterior neck scar contracture reconstruction (Fig. 9) [19].

### Free flaps

**Fig. 10:**
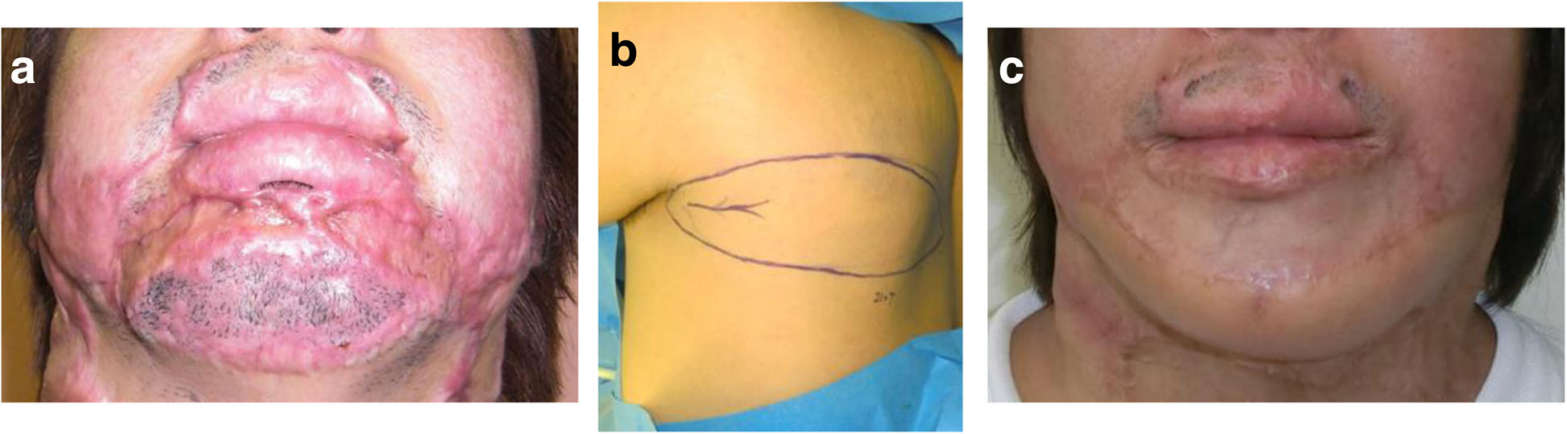
Reconstruction of chin scars by using a free scapular flap. **a** Preoperative view. **b** Design of the free scapular flap. **c** Thirty-six months after the operation. Free flaps remain a good choice for replacing large or specialized defects because they have good functional or esthetic results and minimal donor-site morbidity

Traditionally, free flaps were used for burn reconstruction when there was exposed bone, tendon, or cartilage present and local or regional flaps were inadequate. Today, the availability of various technologies such as negative pressure wound therapy and dermal substitutes means that free flaps are rarely the only choice in burn reconstruction. Moreover, since free flaps are always harvested as island flaps, their contracture-releasing effect is inferior to that of skin-pedicled flaps. However, free flaps remain a good choice for replacing large or specialized defects because they have good functional or esthetic results and minimal donor-site morbidity (Fig. 10).

Free perforator flaps have the advantage over other flap types in that they can be thinned and associate with little donor-site morbidity. To further reduce donor-site morbidity, anterolateral thigh (ALT) flaps [25] or conventional flaps such as groin flaps and scapular flaps are useful. If a long vascular pedicle is needed, perforator flaps such as ALT flaps, deep inferior epigastric perforator (DIEP) flaps, and conventional flaps such as the radial forearm flap and the latissimus dorsi flap can be used. Most surgeons will be familiar with these flaps.

## Conclusions

When used with the appropriate timing and when selected on the basis of individual factors, surgical techniques can improve mature scars. Those methods include z-plasties, w-plasties, STSG, FTSG, local flaps (including the square flap method and the propeller flap), and expanded flaps, distant flaps, regional flaps, and free flaps.

## Abbreviations

ALT: Anterolateral thigh
DIEP: Deep inferior epigastric perforator
FTSG: Full-thickness skin grafts
IMAP: Internal mammary artery perforator
SCAP: Superficial cervical artery perforator
STSG: Split-thickness skin grafts
TAP: Thoracodorsal artery perforator:
